# Health Benefits from Upgrading Public Buses for Cleaner Air: A Case Study of Clark County, Nevada and the United States

**DOI:** 10.3390/ijerph16050720

**Published:** 2019-02-28

**Authors:** John O. Olawepo, L.-W. Antony Chen

**Affiliations:** Department of Environmental and Occupational Health, School of Community Health Sciences, University of Nevada Las Vegas, Las Vegas, NV 89154, USA; olawepo@unlv.nevada.edu

**Keywords:** transit buses, air pollution, health impact, economic cost, alternative fuel, CNG, diesel

## Abstract

Public transit buses, which move more than 5 billion passengers annually in the United States (U.S.), can contribute substantially to the environmental health burden through emitted air pollutants. As a leader in transforming to cleaner bus fleets, the Regional Transport Commission of Southern Nevada (RTC) has been transitioning from diesel to compressed natural gas (CNG) transit buses since 1999. By 2017, ~75% of RTC’s buses operating in Clark County, Nevada were CNG-powered. This study assesses the health benefits of the venture using the US Environmental Protection Agency’s (EPA) Co-Benefits Risk Assessment (COBRA) model, considering the emission and exposure changes from the 2017 baseline for two hypothetical scenarios: (1) no transition (CC_D) and (2) complete transition (CC_N). The CC_D scenario shows realized health benefits, mostly due to avoided mortality, of $0.79–8.21 million per year for 2017 alone, while CC_N suggests an additional $0.88–2.24 million annually that could be achieved by completing the transition. The wide range of estimates partly reflects uncertainties in determining diesel bus emissions under business-as-usual. These health benefits were not limited locally, with ~70% going to other counties. Two national-scale scenarios, US_D and US_N, were also constructed to explore the health impact of transitioning from diesel to CNG buses across the U.S. As of 2017, with CNG powering only ~20% of transit bus mileages nationwide, there could be massive unrealized health benefits of $0.98–2.48 billion per year including 114–258 avoided premature deaths and >5000 avoided respiratory and cardiovascular illnesses. Taking into account the health benefits, economic costs, and the inter-state nature of air pollution, expanding federal assistances to accelerate a nationwide transition to cleaner bus fleets is highly recommended.

## 1. Introduction

According to the World Health Organization (WHO) [1], air pollution causes more mortality and loss of disability-adjusted life years (DALYs) than any other environmental health issues globally. Motor vehicle emissions contribute substantially to urban air pollution around the world [2,3] and have been a target for mitigation. While diesel and gasoline remain the dominant transportation fuels used today, newer technologies, such as electric, hybrid, liquefied petroleum gas (LPG), compressed natural gas (CNG), and hydrogen-powered vehicles, are shown to be cleaner options [4]. For public health and sometimes economic considerations, urban mass transportation systems such as transit buses are often the first to adopt these technologies with public investment. The health benefits of transitioning from conventional to cleaner fuels are rarely assessed when carrying out specific transition programs. However, the impact could be profound. A recent assessment by Dey et al. [5] shows that a complete ban of diesel traffic in Dublin, Ireland and replacing it with CNG and electric vehicles by 2025 will result in a saving of 300 DALYs and €43.8 million per year.

As of 2015, there are about 1.3 billion vehicles globally, with the United States (U.S.) having the world’s largest fleet [4]. In 2015, public transit was responsible for about 10.5 billion unlinked passenger trips in the U.S. and 50% of these passenger trips were by transit buses [6]. The number of fixed-route and demand–response transit buses in the US was estimated to be just above 140,000, with about half of them being diesel-powered [6]. Compared to 1995 where diesel buses made up 95% of transit buses, this demonstrates a remarkable transition over the past 20 years. Despite the improvements in transit buses’ use of cleaner fuel, they still contribute to air pollution. Air pollution due to all on-road vehicles in the U.S. has been estimated to cause ~15,000 premature deaths annually [7], but those due to transit buses are unknown.

Clark County, Nevada, where Las Vegas is located, has a population of over 2 million and a growth rate of 2.2% in 2015. Due to a relatively high population density, air pollution from on-road vehicle emissions is a major health concern [8]. Green et al. [9] attributed >40% of PM_2.5_ (airborne particulate matter with aerodynamic diameter less than 2.5 µm, one of the six criteria air pollutants regulated by the US Environmental Protection Agency (EPA)) to motor vehicle exhausts. In Clark County, the Regional Transport Commission of Southern Nevada (RTC) is responsible for public transit. As of 2015, RTC was ranked the 26th largest transit agency in the US, with 66,856,000 unlinked passenger trips per year [6]. Starting in 1999, RTC instituted a program of gradually replacing transit buses (then powered by diesel or gasoline engines) that reach the end of life (12 years or 500,000 miles) with new buses powered by CNG to help mitigate urban air pollution [10].

As of July 2017, RTC had 747 transit buses in maximum service of which 76.8% (*n* = 574) were CNG buses, 16.5% (*n* = 123) were diesel buses, and 6.7% (*n* = 50) were diesel–electric hybrid buses. The aims of this study are to: (a) assess the impact of RTC’s bus transition from diesel to CNG fuels on the health and wellbeing of the population through improved air quality; and (b) explore the potential national impact if all U.S. counties implement similar programs. The air quality and health assessment are based on the National Emission Inventory (NEI) [11] and Co-Benefits Risk Assessment (COBRA) health impacts screening and mapping tool [12,13] developed by U.S. EPA with 2017 as the baseline year. The calculated health benefits in economic value are discussed alongside the life cycle cost of CNG versus diesel buses and current level of federal assistance to transition programs across the U.S. Conclusions from this study offer a justification to further public investment in cleaner bus fleets at both regional and national levels.

## 2. Methods and Data

### 2.1. COBRA Model

The COBRA screening tool is an integrated model for evaluating the impact of cleaner energy policies that will lead to better air quality with outputs that are readily used by policymakers [12]. It adopts a 3-step procedure: (1) exposure assessment; (2) estimation of health impact associated with the exposure; and (3) estimation of economic (i.e., dollar) value of the health impacts as described in Hou et al. [14] and Thomson et al. [15]. All assessments are based on PM_2.5_, in accordance with the WHO’s approach for evaluating the global burden of disease due to air pollution [1,16]. 

As a first step, COBRA (version 3.2) links sources of PM_2.5_ and precursors (i.e., sulfur dioxide (SO_2_), reactive nitrogen oxides (NO_x_), ammonia (NH_3_), and volatile organic compounds (VOC)) to annual PM_2.5_ concentrations by U.S. county for 2017 or 2025 via a source–receptor (S–R) matrix. The S–R matrix stems from more sophisticated air quality models which simulate both the dispersion of primary PM_2.5_ and formation of secondary PM_2.5_ from the precursors. With the S–R matrix, the COBRA model can estimate changes in PM_2.5_ concentrations across the U.S. due to the emission reduction (or increase) from individual source categories.

Next, COBRA estimates the health risk altered by the changing PM_2.5_ concentrations. In this step the prescribed dose-response curves for 12 health endpoints (i.e., adult and infant mortality, non-fatal heart attacks, respiratory- and cardiovascular-related hospitalizations, acute bronchitis, upper and lower respiratory symptoms, asthma-related emergency room visits, asthma exacerbations, minor restricted activity days, and work days lost due to illness) are used. In the final step, economic values of the health effects in 2017 dollars, with the high and low estimates, are determined by county using a discount rate of 3% or 7%. The 3% discount rate simulates a more conservative economic growth forecast and 7% simulates a more progressive forecast for the next decade.

### 2.2. Clark County Bus Emission Scenarios

Emission changes due to the transition of RTC buses were considered in the COBRA model. Table 1 presents the emissions from transit buses in Clark County, Nevada based on the most recent NEI data available for 2014. Including both the fixed-route and core paratransit services, RTC had 706 buses with a total vehicle mile traveled (VMT) of 31.2 million in 2014. The number of buses and VMT increased to 747 and 33.8 million, respectively, in 2017. It should be noted that all gasoline buses were upgraded to CNG buses between 2014 and 2017 [17]. Based on fuel-specific VMT, the 2014 emissions were scaled up for 2017 (Table 1) to be compatible with the COBRA 2017 baseline. The scale-up assumes the same average emission factors (EFs) between 2014 and 2017 for RTC’s diesel or CNG buses. This is rationalized as new diesel buses with substantially lower emissions had not been brought into service during the three years and new CNG buses (model year 2015 or later) perform similarly to existing CNG buses (model years 2012–2014) with respect to emissions of CO, NO_x_, PM_2.5_, and VOC [18].

Two scenarios for Clark County (CC_D and CC_N) were constructed. The CC_D scenario assumes all the buses were diesel-fueled in 2017. It represents business as usual (BAU) without investing in the bus transition program since 1999, providing that conventional diesel buses had long been considered more cost effective than gasoline or CNG transit buses if not accounting for negative externalities [19]. On the contrary, CC_N assumes an aggressive program that completes the transition to all CNG-powered buses by 2017. Thus CC_D and CC_N attribute all 2017 VMT in Table 1 to diesel and CNG buses, respectively.

The emission increases under CC_D result from higher diesel emission factors than CNG emission factors. However, in this scenario newer diesel buses would have been utilized by RTC, yielding average EFs lower than the actual values in 2014–2017. The practical ranges of diesel bus EFs are summarized in Supplementary Materials Table S1, from which the upper- and lower-bound emission changes for CC_D are estimated and presented in Table 2. On the other hand, the same EFs (i.e., 2014–2017 averages) are assumed for all existing and new CNG transit buses under CC_N; the emission reductions resulting from attributing all diesel VMT to CNG are also shown in Table 2.

Table 2 served as inputs for COBRA to estimate the air quality and health effects. Finally, we aggregated the health effects from CC_D and CC_N to determine the total benefits that could be achieved by the diesel-to-CNG transition. 

### 2.3. U.S. Bus Emission Scenarios

The average annual VMT for a RTC bus is ~45,000, substantially higher than the U.S. average of 34,053 for fixed-route and 23,576 for paratransit/demand-response buses [20]. Providing that in 2017, there were 71,299 and 65,416 fixed-route and on-demand service buses, respectively, across the U.S. [6], VMT for all the U.S. transit buses was estimated to be 3.97 billion for 2017. While the breakdown of fuel-specific VMT varied by region, for the first-order estimate, we attributed 60%, 20%, and 20% of the VMT to diesel, CNG, and gasoline, respectively, for 2017, consistent with the fuel consumption breakdown from fixed-route and demand-response buses combined [6]. Using the average EFs of Clark County 2014–2017, this VMT breakdown would estimate a total emission of 1530 tons primary PM_2.5_, 62.8 tons SO_2_, 51,500 tons NO_x_, 110 tons NH_3_, and 4170 tons VOC from transit buses nationwide.

Two national-scale scenarios, US_D and US_N, were explored for changes in the emissions of PM_2.5_ and precursors. Like the RTC scenarios, US_D assumes that all transit buses had been powered by diesel fuel and US_N assumes them powered entirely by CNG in 2017. For US_D, changes in emissions should depend on the composition of the current, non-diesel and hypothetical, diesel bus fleets, which likely vary by state and by county. Without county-specific information, the high and low diesel EFs in Supplementary Materials Table S1 were used to estimate the range of emission changes for the U.S. as a whole. The emission reductions under US_N were estimated, like CC_N, using the same CNG EFs in Supplementary Materials Table S1.

National emission changes under US_D and US_N are also summarized in Table 2, based on which COBRA was run to assess the air quality health effects. All assessments include both the 3% and 7% discount rates. The county-level emission changes and health outcomes from COBRA would be more uncertain than the national results. However, this analysis helps identify regions more impacted by emission reductions. 

## 3. Results

**Figure 1:**
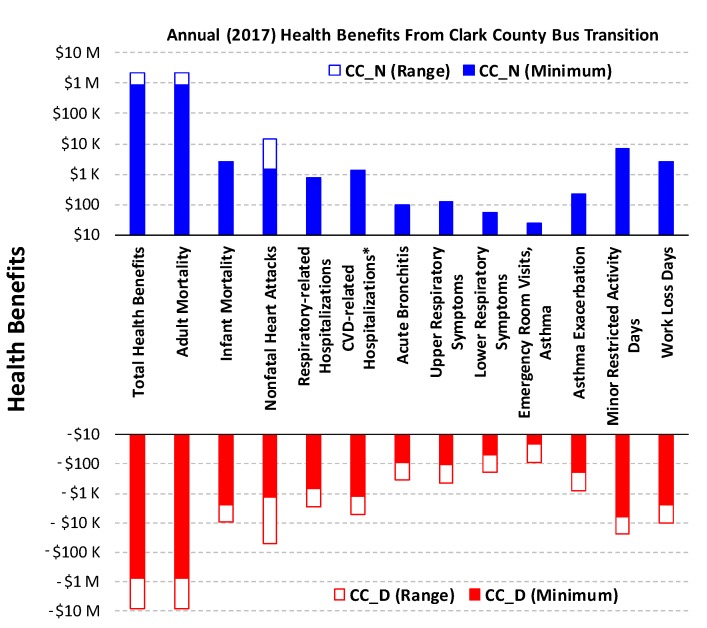
Annual health benefits in economic cost under CC_D and CC_N. The minimum and range are determined from the lower- and upper-bound estimates with both 3% and 7% discount rate (Supplementary Materials Tables S2–S4). M: millions. K: thousands. * Excluding heart attacks.

**Figure 2:**
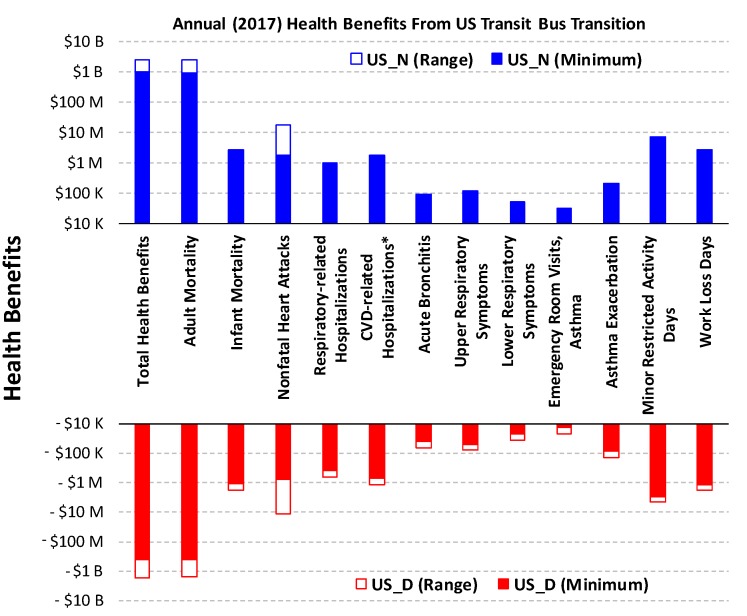
Annual health benefits in economic cost under US_D and US_N. The minimum and range are determined from the lower- and upper-bound estimates with both 3% and 7% discount rate (Supplementary Materials Tables S5–S7). B: Billions: M: millions. K: thousands. * Excluding heart attacks.

The health benefits corresponding to the four scenarios, as estimated by COBRA, are summarized in Supplementary Materials Tables S2–S7 and also compared in Figure 1 and Figure 2. For CC_D, the loss of health benefits ranges from $0.79 million to $8.21 million per year, mostly due to additional adult and infant mortality, non-fatal heart attacks, and minor restricted activity days associated with increased PM_2.5_ exposure among all US counties. This can be viewed as the “realized” health benefits from the bus transition program, and the wide range of estimates reflects the uncertainty in the age distribution of diesel bus fleet under the BAU (no transition) scenario, as well as other uncertainties in the COBRA model. The CC_N would result in additional health benefits of $0.88–2.24 million per year. Therefore, the maximum health benefits from the RTC bus transition program (CC_D→N) is $1.67–10.5 million per year, including prevention of up to 2 premature deaths, 1 non-fatal heart attack, 7–16 asthma exacerbation events in children aged 6 to 18 years, 168–413 minor restricted activity days, and 29–70 days of lost work (Supplementary Materials Tables S2–S4).

The US_D scenario shows a $0.39–1.63 billion loss of health benefits per year, while the US_N scenario would produce total health benefits ranging from $0.978 billion to $2.48 billion per year. For both scenarios the economic impact is mostly attributed to the changes in adult mortality (Supplementary Materials Tables S5–S7). When combined (US_D→N), an overall health benefit of $1.37–4.11 billion per year may be achieved by transitioning all U.S. transit bus fleets from diesel to CNG. The national outcome exceeds four-hundred times that from Clark County’s effort alone and avoids up to 160–428 premature deaths, saving up to 20–213 non-fatal heart attacks, 4770–5630 asthma exacerbation, 131,000–155,000 minor restricted activity days, and 22,100–26,100 days of lost work per year. This assessment also reveals that 1–3% of traffic-pollution-related premature deaths in the U.S. (15,000 per year) may be attributed to transit buses.

**Figure 3:**
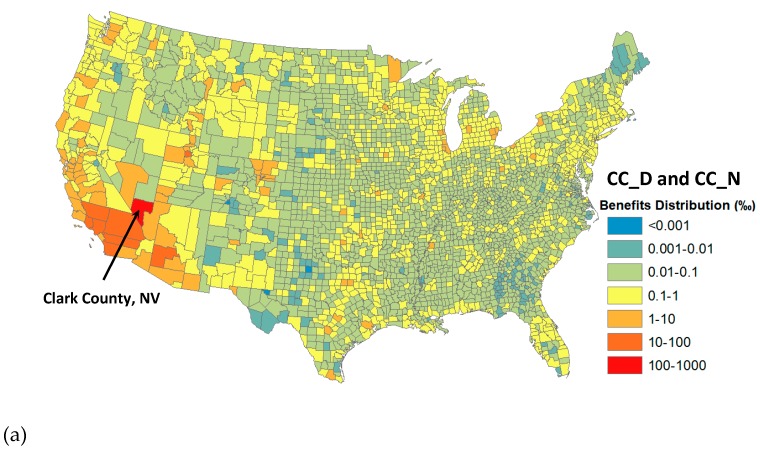

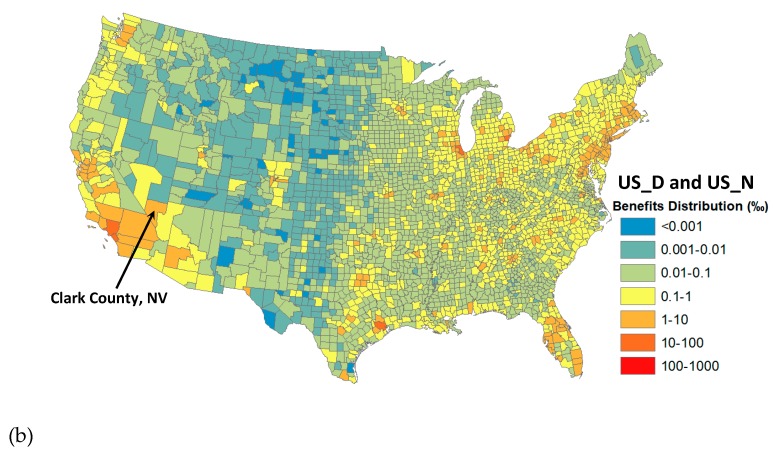
Distribution of health benefits (in ‰) from reducing transit bus emissions (**a**) in Clark County, Nevada and (**b**) all over the US, based on the COBRA modeling for (a) CC_D and CC_N and (**b**) US_D and US_N scenarios. All CC (or US) scenarios yield consistent spatial distributions (see Supplementary Materials Tables S8 and S9 for data).

The health benefits are not equally shared among the states and counties. Clark County could capture ~30% of the benefits, or $0.50–3.14 million per year, from reducing its transit bus emissions (i.e., CC_D→N), while the remaining benefits are distributed mostly to Southern California/Arizona counties owing to their proximity and large population bases (Figure 3a). When emission reductions occur throughout the U.S., larger metropolitan areas, such as Cook County in Illinois, Los Angeles and Orange County in California, Wayne County in Michigan, and Harris County in Texas, enjoy more health benefits than rural counties with lower populations (Figure 3b). In the case of US_D→N, Clark County’s share of benefits is ~0.12% or $1.64–4.93 million per year which appears to be higher than the estimate of $0.50–3.14 million from the Clark County-only emission reductions. It suggests that Clark County can benefit substantially from emission reductions in other U.S. counties.

## 4. Discussion

Our findings (CC_D) show that the RTC’s venture of switching to cleaner fuels for running its transit bus fleet has produced health benefits of $0.234–2.46 million for Clark County citizens in 2017 alone. Additional benefits of $0.266–0.677 million might have been achieved by completing the transition before 2017 (CC_N). The annual health benefits could be larger for years prior to 2017 when RTC replaced diesel buses with higher EFs than those in 2017. Assuming a bus service life of 12 years (500,000 miles) regardless of technology, Lajunen and Lipman [21] estimate a life cycle cost (LCC) of $0.815–0.875 million and $0.780–0.838 million for diesel and CNG transit buses in the US, respectively, for 2015. In Europe, the LCC is €0.579–0.590 million (diesel) and €0.658–0.663 million (CNG). The US and Europe LCCs differ most in the price of diesel and natural gas fuels between the two markets. Nonetheless, with a lower natural gas price in the U.S. today, CNG-powered transit buses are competitive with diesel buses on the LCC basis and become an economic choice when accounting for the air quality and health benefits.

It should be noted that health benefits from emission reduction are not limited locally. The output from Clark County, according to CC_D and CC_N, is more than twice those realized within the county. Clark County has also benefited from transit bus upgrades in other regions. This underscores the importance of cooperation among the states and planning at the federal level when undertaking this venture. The major barrier in the transition is the capital expenses, including new CNG buses or retrofits and fueling/maintenance infrastructure, incurred by local transportation agencies. Federal financial assistances can substantially lower the agencies’ burden and catalyze the transition [22]. For the fiscal year 2017, the U.S. Department of Transportation (U.S. DOT) announced $26.6 million funding for 11 projects to replace older diesel buses with CNG buses or improve CNG fueling facilities and additional $18.7 million towards even cleaner electric buses as part of the Buses and Bus Facilities Infrastructure Investment Program [23]. However, demand for DOT’s assistance to upgrade buses and bus facilities far exceeded available funds. Given the large unrealized health benefits ($0.978–2.48 billion per year according to US_N) associated with transit bus emissions, increasing investment in the DOT’s Bus Program to accelerate the transition to cleaner fuels is highly justified.

Uncertainties in our assessment can result from emission inventories and atmospheric transport model used in COBRA, as well as the assumed dose–outcome relationships [13]. Additionally, COBRA does not consider other vehicle-related air pollutants, particularly ozone (O_3_), which also contribute to respiratory illnesses and mortality [24]. Therefore, the potential impacts reported here may be undervalued.

## 5. Conclusions

The RTC of Southern Nevada started to switch their public transit buses from diesel- to CNG-powered vehicles in 1999, and as at 2017, more than 75% of its bus fleet were already powered by CNG. For the first time, the health benefits of this venture via reducing PM_2.5_ exposure level is assessed with the COBRA model. The benefits, including avoided mortality, respiratory- and cardiovascular-related illnesses and hospitalizations, minor restricted activity days, and work days lost, were valuated at $0.79–8.21 million per year for 2017 alone, with ~30% and 70% distributed in- and out-side Clark County, respectively. The wide range of estimates partly reflects uncertainties in diesel bus EFs in case (business-as-usual) RTC has been acquiring new diesel buses instead of CNG buses. Additional benefits of $0.884–2.24 million per year would have been gained if RTC had completed the transition before 2017. 

Across the U.S., diesel fuel still powered ~60% of VMT from all transit buses (~20% by gasoline) in 2017, suggesting massive unrealized health benefits of $0.978–2.48 billion per year including 114–258 potentially avoided premature deaths from the diesel (or gasoline)-to-CNG switch. The benefits go far beyond states or counties where emission reductions are made. Based on the life cycle cost and health benefits, the CNG-powered bus system is a favorable option in the US today, though the transition from diesel to CNG buses is usually delayed due to capital expenses incurred by local transportation agencies. Increasing the current federal investment to address this barrier is highly justified by the gains in US public health.

## Figures and Tables

**Table 1:** Number of buses, vehicle mile traveled (VMT), and emissions (tons/year) of the RTC buses by fuel type for 2014 and 2017.

Year and Fuel Type *	2014			2017		
	Diesel	CNG	Gasoline	Diesel	CNG	Gasoline
Number of Buses	317	180	209	173	574	0
Million VMT	13.0	10.1	8.06	7.25	26.5	0
PM_2.5_ (Primary)	8.23	0.0725	0.156	4.59	0.190	0
SO_2_	0.302	0.0123	0.0656	0.168	0.0323	0
NO_x_	277	4.00	4.41	154	10.5	0
NH_3_	0.517	0.0607	0.109	0.288	0.159	0
VOC	21.8	0.632	1.35	12.1	1.65	0

* Bus and VMT information were acquired from RTC. Emissions for 2014 were calculated using the U.S. EPA Motor Vehicle Emission Simulator (MOVES) model and had been reported to NEI, while emissions for 2017 were scaled up from the 2014 emissions based on fuel-specific VMT and average emission factors. All emissions are in tons/year. CNG: compressed natural gas; VMT: vehicle miles traveled; RTC: The Regional Transport Commission of Southern Nevada; EPA: the U.S. Environmental Protection Agency; NEI: The National Emission Inventory.

**Table 2:** Emission changes (tons/year) from the 2017 baseline for four scenarios evaluated in this study and used as the COBRA model inputs.

Scenario *	CC_D		CC_N ^a^	US_D		US_N ^b^
	Upper ^c^	Lower ^d^		Upper ^e^	Lower ^f^	
PM_2.5_ (Primary)	16.58	3.87	−4.53	983.98	595.98	−1500.13
SO_2_	0.58	0.31	−0.16	29.48	21.02	−57.96
NO_x_	554.0	138.6	−151.0	33,082.6	20,397.9	−49,924.0
NH_3_	0.89	0.30	−0.24	47.64	29.39	−86.34
VOC	42.70	9.87	−11.67	2474.72	1472.67	−3921.06

^a^ CC_N (complete transition) assumes that all diesel transit buses in Clark County (21.5% VMT) are substituted by CNG buses. ^b^ US_N (complete transition) assumes that all diesel and gasoline transit buses in the U.S. (80% VMT) are substituted by CNG buses. ^c^ The upper bound under CC_D (no transition) assumes that all CNG transit buses (78.5% VMT) are substituted by older diesel buses with “high” average emission factors (EFs) (Supplementary Materials Table S1). ^d^ The lower bound under CC_D assumes that all CNG transit buses (78.5% VMT) are substituted by newer diesel buses with “low” average EFs (Supplementary Materials Table S1). ^e^ The upper bound under US_D (no transition) assumes that all CNG and gasoline transit buses in the U.S. (40% VMT) are substituted by older diesel buses with “high” average EFs (Supplementary Materials Table S1). ^f^ The lower bound under US_D assumes that all CNG and gasoline transit buses in the U.S. (40% VMT) are substituted by newer diesel buses with “low” average EFs (Supplementary Materials Table S1). * For COBRA (Co-Benefits Risk Assessment) modeling, emission changes in CC_D and CC_N were applied to Clark County, NV, while emission changes in US_D and US_N were applied to the US 2017 emission inventory.

## Supplementary Materials

The following are available online at https://www.mdpi.com/1660-4601/16/5/720/s1, Table S1: Emission factors (g/mile) of diesel, gasoline, and CNG transit buses used in this assessment, Table S2: Estimated annual health impacts and associated economic costs from the COBRA model, if all CNG transit buses in Clark County, Nevada were replaced with “older” diesel buses in 2017 (CC_D scenario, upper bound), Table S3: Estimated annual health impacts and associated economic costs from the COBRA model, if all CNG transit buses in Clark County, Nevada were replaced with “newer” diesel buses in 2017 (CC_D scenario, lower bound), Table S4: Estimated annual health impacts and associated economic costs from the COBRA model, if all diesel transit buses in Clark County, Nevada were replaced with CNG buses in 2017 (CC_N scenario), Table S5: Estimated annual health impacts and associated economic costs from the COBRA model, if all CNG and gasoline transit buses in the U.S. were replaced with “older” diesel buses in 2017 (US_D scenario, upper bound), Table S6: Estimated annual health impacts and associated economic costs from the COBRA model, if all CNG and gasoline transit buses in the U.S. were replaced with “newer” diesel buses in 2017 (US_D scenario, lower bound), Table S7: Estimated annual health impacts and associated economic costs from the COBRA model, if all diesel and gasoline transit buses in the U.S. were replaced with CNG buses in 2017 (US_N scenario), Table S8: Distribution of health benefits (in $ amount and in ‰) by county under the CC_D and CC_N scenarios, Table S9: Distribution of health benefits (in $ amount and in ‰) by county under the US_D and US_N scenarios.
