# Supplementary material for: Health Benefits from Upgrading Public Buses for Cleaner Air: A Case Study of Clark County, Nevada and the United States
Source: Int J Environ Res Public Health. 2019 Feb 28;16(5):720. doi: 10.3390/ijerph16050720 (PMC6427397; doi:10.3390/ijerph16050720)
Supplement: Supplementary file 1 [file ijerph-16-00720-s001.zip › Olawepo&Chen2019COBRA Paper_v3 2-11-2019-suppl.pdf]

Health benefits from upgrading public buses for cleaner air: A case study of Clark County, Nevada and the United States

John O. Olawepo[1] and L.-W. Antony Chen[1]

[1]*Department of Environmental and Occupational Health, School of Community Health Sciences, University of Nevada Las Vegas, Las Vegas, NV, 89154*

Supplementary Information

Table S1. Emission factors (g/mile) of diesel, gasoline, and CNG transit buses used in this assessment.

	Diesel		Gasoline*	CNG*
	High*	Low**		
$PM_{2.5}$ (Primary)	0.57	0.020	0.018	0.0065
SO_2	0.021	0.009	0.007	0.001
NO_x	19.3	1.21	0.50	0.36
NH_3	0.036	0.010	0.012	0.0054
VOC	1.51	0.087	0.15	0.057

*Average EFs derived from fuel-specific transit bus emissions for Clark County, Nevada (NEI 2014) and numbers based on RTC records.

**Average EFs for newer diesel transit buses (model years 2015–2017) (Cai et al., 2013).

Reference

Cai, H., Burnham, A., & Wang, M. (2013). *Updated emission factors of air pollutants from vehicle operations in GREET^TM^ using MOVES*. Systems Assessment Section, Energy Systems Division, Argonne National Laboratory. Available from: https://greet.es.anl.gov/publication-vehicles-13 (Accessed on 2/10/2019)

Table S2. Estimated annual health impacts and associated economic costs from the COBRA model, if all CNG transit buses in Clark County, Nevada were replaced with “older” diesel buses in 2017 (CC_D scenario, upper bound). See Table 2 for COBRA input.

**Effect Description**	**Negative Health Impacts**	**Economic Benefits ($) (All U.S.)**	
		**3% discount**	**7% discount**
Total Health Benefits (low estimate)		-3,626,606	-3,236,525
Total Health Benefits (high estimate)		-8,208,132	-7,321,095
**Mortality**			
Adult Mortality (low estimate)	0.4	-3,566,951	-3,177,027
Adult Mortality (high estimate)	0.9	-8,101,206	-7,215,615
Infant Mortality	<0.1	-9,756	-9,756
**Cardiac Effects**			
Nonfatal Heart Attacks (low estimate)	<0.1	-5,701	-5,545
Nonfatal Heart Attacks (high estimate)	0.4	-52,971	-51,526
Hospital Admits, Cardiovascular (except heart attacks)	0.1	-5,077	-5,077
**Respiratory Effects**			
Hospital Admits, All Respiratory	0.1	-2,819	-2,819
Acute Bronchitis	0.7	-357	-357
Upper Respiratory Symptoms	12.1	-449	-449
Lower Respiratory Symptoms	8.5	-199	-199
Emergency Room Visits, Asthma	0.2	-87	-87
Asthma Exacerbation	12.5	-802	-802
**Effect on Economic Activity**			
Minor Restricted Activity Days	323.8	-24,608	-24,608
Work Loss Days	54.7	-9,801	-9,801

Table S3. Estimated annual health impacts and associated economic costs from the COBRA model, if all CNG transit buses in Clark County, Nevada were replaced with "newer" diesel buses in 2017 (CC_D scenario, lower bound). See Table 2 for COBRA input.

**Effect Description**	**Negative Health Impacts**	**Economic Benefits ($) (All U.S.)**	
		**3% discount**	**7% discount**
Total Health Benefits (low estimate)		-887,255	-791,821
Total Health Benefits (high estimate)		-2,008,102	-1,791,090
**Mortality**			
Adult Mortality (low estimate)	<0.1	-872,667	-777,270
Adult Mortality (high estimate)	0.2	-1,981,940	-1,765,282
Infant Mortality	<0.1	-2,386	-2,386
**Cardiac Effects**			
Nonfatal Heart Attacks (low estimate)	<0.1	-1,396	-1,358
Nonfatal Heart Attacks (high estimate)	0.1	-12,969	-12,615
Hospital Admits, Cardiovascular (except heart attacks)	<0.1	-1,243	-1,243
**Respiratory Effects**			
Hospital Admits, All Respiratory	<0.1	-691	-691
Acute Bronchitis	0.2	-87	-87
Upper Respiratory Symptoms	3.0	-110	-110
Lower Respiratory Symptoms	2.1	-49	-49
Emergency Room Visits, Asthma	<0.1	-21	-21
Asthma Exacerbation	3.1	-196	-196
**Effect on Economic Activity**			
Minor Restricted Activity Days	79.1	-6,014	-6,014
Work Loss Days	13.4	-2,395	-2,395

Table S4. Estimated annual health impacts and associated economic costs from the COBRA model, if all diesel transit buses in Clark County, Nevada were replaced with CNG buses in 2017 (CC_N scenario). See Table 2 for COBRA input.

**Effect Description**	**Positive Health Impacts**	**Economic Benefits ($) (All U.S.)**	
		**3% discount**	**7% discount**
Total Health Benefits (low estimate)		990,911	884,328
Total Health Benefits (high estimate)		2,242,734	2,000,366
**Mortality**			
Adult Mortality (low estimate)	0.1	974,612	868,071
Adult Mortality (high estimate)	0.2	2,213,518	1,971,545
Infant Mortality	<0.1	2,666	2,666
**Cardiac Effects**			
Nonfatal Heart Attacks (low estimate)	<0.1	1,558	1,515
Nonfatal Heart Attacks (high estimate)	0.1	14,474	14,079
Hospital Admits, Cardiovascular (except heart attacks)	<0.1	1,387	1,387
**Respiratory Effects**			
Hospital Admits, All Respiratory	<0.1	770	770
Acute Bronchitis	0.2	97	97
Upper Respiratory Symptoms	3.3	123	123
Lower Respiratory Symptoms	2.3	54	54
Emergency Room Visits, Asthma	<0.1	24	24
Asthma Exacerbation	3.4	219	219
**Effect on Economic Activity**			
Minor Restricted Activity Days	88.5	6,723	6,723
Work Loss Days	14.9	2,678	2,678

Table S5. Estimated annual health impacts and associated economic costs from the COBRA model, if all CNG and gasoline transit buses in the U.S. were replaced with “older” diesel buses in 2017 (US_D scenario, upper bound). See Table 2 for COBRA input.

**Effect Description**	**Negative Health Impacts**	**Economic Benefits ($) (All U.S.)**	
		**3% discount**	**7% discount**
Total Health Benefits (low estimate)		-719,432,456	-642,035,630
Total Health Benefits (high estimate)		-1,627,266,956	-1,451,470,679
**Mortality**			
Adult Mortality (low estimate)	74.7	-707,690,862	-630,328,992
Adult Mortality (high estimate)	169.5	-1,605,177,581	-1,429,706,133
Infant Mortality	0.2	-1,806,267	-1,806,267
**Cardiac Effects**			
Nonfatal Heart Attacks (low estimate)	9.1	-1,247,836	-1,212,880
Nonfatal Heart Attacks (high estimate)	84.5	-11,595,617	-1,1270,787
Hospital Admits, Cardiovascular (except heart attacks)	26.9	-1,173,951	-1,173,951
**Respiratory Effects**			
Hospital Admits, All Respiratory	21.6	-654,274	-654,274
Acute Bronchitis	118.7	-63,497	-63,497
Upper Respiratory Symptoms	2,160.5	-80,031	-80,031
Lower Respiratory Symptoms	1,512.8	-35,423	-35,423
Emergency Room Visits, Asthma	44.7	-21,385	-21,385
Asthma Exacerbation	2,228.8	-143,379	-143,379
**Effect on Economic Activity**			
Minor Restricted Activity Days	61,372.2	-4,664,560	-4,664,560
Work Loss Days	10,325.6	-1,850,992	-1,850,992

Table S6. Estimated annual health impacts and associated economic costs from the COBRA model, if all CNG and gasoline transit buses in the U.S. were replaced with "newer" diesel buses in 2017 (US_D scenario, lower bound). See Table 2 for COBRA input.

Effect Description	Negative Health Impacts	Economic Benefits ($) (All U.S.)	
		3% discount	7% discount
Total Health Benefits (low estimate)		-440,624,010	-393,221,451
Total Health Benefits (high estimate)		-996,628,637	-888,961,186
**Mortality**			
Adult Mortality (low estimate)	45.8	-433,433,242	-386,052,092
Adult Mortality (high estimate)	103.8	-983,100,277	-875,631,776
Infant Mortality	0.1	-1,106,456	-1,106,456
**Cardiac Effects**			
Nonfatal Heart Attacks (low estimate)	5.6	-764,271	-742,861
Nonfatal Heart Attacks (high estimate)	51.8	- 7,101,863	-6,902,913
Hospital Admits, Cardiovascular (except heart attacks)	16.5	-718,917	-718,917
**Respiratory Effects**			
Hospital Admits, All Respiratory	13.2	-400,692	-400,692
Acute Bronchitis	72.7	-38,887	-38,887
Upper Respiratory Symptoms	1,323.2	-49,015	-49,015
Lower Respiratory Symptoms	926.5	-21,694	-21,694
Emergency Room Visits, Asthma	27.4	-13,091	-13,091
Asthma Exacerbation	1,365.0	-87,810	-87,810
**Effect on Economic Activity**			
Minor Restricted Activity Days	37,582.6	-2,856,442	-2,856,442
Work Loss Days	6,323.1	-1,133,494	-1,133,494

Table S7. Estimated annual health impacts and associated economic costs from the COBRA model, if all diesel and gasoline transit buses in the U.S. were replaced with CNG buses in 2017 (US_N scenario). See Table 2 for COBRA input.

Effect Description	Positive Health Impacts	Economic Benefits ($) (All U.S.)	
		3% discount	7% discount
Total Health Benefits (low estimate)		1,096,324,170	978,382,734
Total Health Benefits (high estimate)		2,479,551,179	2,211,682,702
**Mortality**			
Adult Mortality (low estimate)	113.9	1,078,417,794	960,529,572
Adult Mortality (high estimate)	258.3	2,445,884,259	2,178,510,196
Infant Mortality	0.3	2,749,948	2,749,948
**Cardiac Effects**			
Nonfatal Heart Attacks (low estimate)	13.9	1,900,932	1,847,717
Nonfatal Heart Attacks (high estimate)	128.8	17,661,475	17,167,061
Hospital Admits, Cardiovascular (except heart attacks)	41.0	1,787,125	1,787,125
**Respiratory Effects**			
Hospital Admits, All Respiratory	32.9	996,179	996,179
Acute Bronchitis	181.1	96,892	96,892
Upper Respiratory Symptoms	3,297.4	122,145	122,145
Lower Respiratory Symptoms	2,308.5	54,054	54,054
Emergency Room Visits, Asthma	68.0	32,548	32,548
Asthma Exacerbation	3,401.5	218,822	218,822
**Effect on Economic Activity**			
Minor Restricted Activity Days	93,699.8	7,121,607	7,121,607
Work Loss Days	15,765.2	2,826,125	2,826,125

Table S8. Distribution of health benefits (in $ amount and in ‰) by county under the CC_D and CC_N scenarios.

*See attached CC&US_Benefits_Distribution.xlsx

Table S9. Distribution of health benefits (in $ amount and in ‰) by county under the US_D and US_N scenarios.

*See attached CC&US_Benefits_Distribution.xlsx
